# Role of Spasticity Severity in the Balance of Post-stroke Patients

**DOI:** 10.3389/fnhum.2021.783093

**Published:** 2021-12-10

**Authors:** Ashraf Mahmoudzadeh, Noureddin Nakhostin Ansari, Soofia Naghdi, Ehsan Ghasemi, Omid Motamedzadeh, Brandon S. Shaw, Ina Shaw

**Affiliations:** ^1^Musculoskeletal Research Center, Isfahan University of Medical Sciences, Isfahan, Iran; ^2^Department of Physiotherapy, School of Rehabilitation, Tehran University of Medical Sciences, Tehran, Iran; ^3^Research Center for War-Affected People, Tehran University of Medical Sciences, Tehran, Iran; ^4^Department of Human Movement Science, University of Zululand, Kwazulu-Natal, South Africa

**Keywords:** stroke, dynamic balance, postural sway, spasticity severity, balance confidence

## Abstract

**Background:** Lower limb spasticity after stroke is common that can affect the balance, increase the risk of falling, and reduces the quality of life.

**Objective:** First, evaluate the effects of spasticity severity of ankle plantar flexors on balance of patients after stroke. Second, to determine the relationship between the spasticity severity with ankle proprioception, passive ankle dorsiflexion range of motion (ROM), and balance confidence.

**Methods:** Twenty-eight patients with stroke based on the Modified Modified Ashworth Scale (MMAS) were divided into two groups: High Spasticity Group (HSG) (MMAS > 2) (*n* = 14) or a Low Spasticity Group (LSG) (MMAS ≤ 2) (*n* = 14). The MMAS scores, Activities-Specific Balance Confidence Questionnaire, postural sway of both affected and non-affected limbs under the eyes open and eyes closed conditions, timed up and go (TUG) test, passive ankle dorsiflexion ROM, and ankle joint proprioception were measured.

**Results:** The ankle joint proprioception was significantly better in the LSG compared to the HSG (*p* = 0.01). No significant differences were found between the LSG and HSG on all other outcome measures. There were no significant relationships between the spasticity severity and passive ankle dorsiflexion ROM, and balance confidence.

**Conclusion:** The severity of ankle plantar flexor spasticity had no effects on balance of patients with stroke. However, the ankle joint proprioception was better in patients with low spasticity. Our findings suggest that the balance is affected regardless of the severity of the ankle plantar flexor spasticity in this group of participants with stroke.

## Introduction

Stroke is a common cause of disability and residual physical impairments following a stroke and can pose a significant threat to quality of life (World Health Organization, 2018). Every year, 3.7 million individuals globally suffer a hemorrhagic stroke, while 7.3 million suffer from an ischemic stroke (Feigin et al., 2017). Specifically, the sensorimotor and cognitive impairments following a stroke can have serious impacts on independence and activities of daily living (ADL) (Geurts et al., 2005). Of these stroke complications, impaired balance is critical for safe mobility, and any deficiencies in balance negatively affect gait, limit ADLs, and/or increases the risk of individuals falling (Paillex and So, 2005; Kollen et al., 2006; Van de Port et al., 2006).

Spasticity is one of common complications of stroke that negatively affects balance. Spasticity is a common sensorimotor disorder defined neurophysiologically as a velocity-dependent increase in muscle tone and stretch reflex hypersensitivity (Lance, 1980). It has been reported as many as 50% of patients with stroke have muscle spasticity (Dorňák et al., 2019). Spasticity contributes in balance dysfunction through various mechanisms (Sinkjær, 1996; Carpenter et al., 2004; Bensoussan et al., 2007; Trumbower et al., 2010). After stroke, lower limb spasticity decreases the range of motion (ROM) and increases the stiffness of the muscles and fascia around the joints (Gao et al., 2011). Further, balance control is further affected when inappropriate muscle and joint afferents and subsequent movement responses occur with inappropriate ankle strategies (Lee et al., 2010).

While lower limb spasticity affects balance, gait, and falling in post-stroke patients (Soyuer and Ozturk, 2007; Sommerfeld et al., 2012), the effects may vary according to the intensity of the muscle spasticity after stroke (Nardone et al., 2001; Cakar et al., 2010; Phadke et al., 2014). To these authors’ knowledge, only one study has investigated the relationship between the spasticity severity and the balance in patients with stroke (Rahimzadeh Khiabani et al., 2017). However, authors assessed only static balance using one force plate, and did not evaluate the ankle proprioception and ROM in patients with stroke. Further, they measured the severity of spasticity based on the Modified Ashworth Scale that its reliability and validity is questioned (Ansari et al., 2006) and caution had been expressed in using it for spasticity assessment (Fleuren et al., 2010). Thus, this study aimed to evaluate the effects of ankle plantar flexor spasticity severity on balance and to determine the relationship between the spasticity severity with ankle proprioception, passive ROM, and balance confidence in post-stroke patients. We hypothesized that stroke subjects with high spasticity would have greater balance impairment compared with stroke subjects with low spasticity.

## Materials and Methods

### Study Design

The protocol of this cross-sectional study has been previously reported (Mahmoudzadeh et al., 2020) with the exception of one modification made on posturography. The present study utilized two force plates to assess the both affected and less affected limbs separately. Modified Modified Ashworth Scale (MMAS) scores, Activities-Specific Balance Confidence Questionnaire, postural sway in the open and closed eyes conditions, timed up and go (TUG) test, ankle dorsiflexion passive ROM, and ankle joint proprioception were measured in two post-stroke patient groups based on the level of ankle plantar flexor spasticity [i.e., High Spasticity Group (HSG) (MMAS > 2) and a Low Spasticity Group (LSG) (MMAS ≤ 2)].

### Setting

The measurements were taken at the Javad Movafaghian Research Center, Tehran, Iran.

### Approval of Study Protocol

The study protocol was approved by the Review Board and the Ethical Committee of the Tehran University of Medical Sciences (IR.TUMS.FNM.REC.1397.012) in compliance with the Helsinki declaration. All participants provided their written informed consent prior to the assessments.

### Informed Consent

All eligible participants provided a written formal consent after receiving information about the research procedure. Study details, risks, and outcome measures were explained to participants prior to giving the written informed consent and taking the measurements.

### Participants

Patients with stroke were included from those who were referred to neurology and physiotherapy clinics in Tehran, Iran. The patients were included if they had the following criteria: (1) unilateral, first-ever Hemorrhagic/Ischemic stroke, (2) ankle plantar flexor spasticity ≥ 1 based on the MMAS, (3) walking ability, (4) no fixed contracture in the ankle, (5) independent standing with eyes open/closed, (6) ability to understand and follow the commands, and (7) no pain in the lower limbs. The exclusion criteria were: (1) vision problems, (2) depression, and (3) taking antispastic medications.

### Sample Size

Considering a previous study and β = Z_β_ = 0.842, α = 0.05, α = Z_α_ = 1.96 (Rahimzadeh Khiabani et al., 2017; Mahmoudzadeh et al., 2020), the sample size was calculated at 28 (*n* = 14 in each group).

### Procedures

The study procedures and measurements utilized in this study have been published previously (Mahmoudzadeh et al., 2020). Demographic data of the all patients were collected prior to the initiation of assessments. All tests were performed by an experienced physiotherapist. Spasticity severity of ankle plantar flexor muscle was evaluated using the MMAS (Ghotbi et al., 2011; Nakhostin Ansari et al., 2012). Patients were classified as High (MMAS ≥ 2) (HSG *n* = 14) and Low (MMAS < 2) (LSG *n* = 14) spasticity. The Activities-Specific Balance Confidence (ABC) questionnaire was used to assess the balance confidence (Salbach et al., 2006; Azad et al., 2016) and includes16 questions asking subjects to score their confidence in performing their activities in daily living from 0% (no confidence) to 100% (complete confidence). Posturography was used to assess the static balance (Sawacha et al., 2013; Lendraitienë et al., 2017) using two force plates which were placed together without spaces between to measure the postural sway of affected and less affected limbs independently. The examiner asked each patient to stand on the force plate with bare feet, heel spacing to be 9 cm, the angle between the two feet being 30 degrees, and upper limbs alongside the body. The patient was asked to look at a point on the wall at a distance of 3 m during the test with an open eye and closed eye. The open or closed eye conditions were randomly applied and a 2-min rest was considered between these two conditions. Each condition was repeated for three times (with intervals of 20 s) and the duration of each repetition was 20 s (Rahimzadeh Khiabani et al., 2017). The dynamic balance of patients was measured using the TUG test (Ng and Hui-Chan, 2005). Ankle passive ROM was measured using a standard goniometer (Radinmehr et al., 2019). The ankle joint proprioception was measured using electrogonometer as reconstruction errors. To assess the proprioception, the average of three repetitions of reconstruction angles measured at the angles of 5° and 15° plantar flexion as well as 15° dorsi flexion were calculated as the angles of reconstruction errors.

### Outcome Measures

The primary outcome measures were the MMAS scores, ABC questionnaire, posturography measures in open- and closed-eyes conditions, and TUG test. The secondary outcome measures were the ankle passive ROM and ankle joint proprioception. BioWare software (Bioware 5.3.2.9-2.0, Kistler Bioware.msi, Kistler Instrument Group) was used for transforming the force plate data numerical mode. Medio-lateral (ML) and antrio-posterior (AP) displacement, average and instant velocity of the center of pressure (COP) and area were calculated using Excel (Excel, 2010, Ink) and MATLAB (MATLAB, R2018b, Ink) softwares.

### Statistical Analysis

Normal distribution of the data was assessed by the Kolmogrov-Smirnov test. *T*-tests were used to compare the clinical data between two groups. A mixed model Repeated Measures ANOVA of 2 × 2 × 2 was performed to analyze the “Group effect” (High spasticity vs. Low spasticity), “Limb effect” (affected and unaffected limbs), “Eye effect” (eyes open and closed conditions), and interactions between variables. The relationships between the severity of spasticity and outcome variables were analyzed using the Spearman’s correlation test. SPSS software (SPSS, version 22, SPSS Inc., Chicago, IL, United States) was used for the data analysis. Statistical significance was defined at α = 0.05.

## Results

### Participant Demographics

Twenty-eight post-stroke patients were included in the current study. High and Low spasticity groups were similar in terms of height, weight, age, the time since the stroke onset, etiology (i.e., ischemic or hemorrhage) and affected side (*P* > 0.05). Demographic characteristics of the two study groups are illustrated in Table 1.

**TABLE 1:** Demographic characteristics of the High (MMAS* ≥ 2) (HSG *n* = 14) and Low (MMAS < 2) (LSG *n* = 14) spasticity study groups.

		Low spasticity group (*n* = 14)	High spasticity group (*n* = 14)	*p*-value
Age (year)		59.14 ± 13.8	54.36 ± 10.02	0.3
Height (centimeters)		164 ± 10.62	168 ± 6.3	0.24
Time since stroke (months)		40.07 ± 26.2	54.92 ± 38.63	0.24
Sex (female) (n)		8	6	0.46
Etiology (ischemic) (n)		11	9	0.41
Affected side	Left (n)	8	6	0.71
	Right (n)	6	8	

Data are presented as means ± SD, *MMAS, Modified Modified Ashworth scale.

### Clinical Measures

No significant difference was found between the two groups for TUG, ABC scores, and ankle passive dorsiflexion ROM (*p* > 0.05). However, ankle joint proprioception was found to be significantly different between the groups (*p* = 0.01) (Table 2).

**TABLE 2:** Clinical characteristics of the High (MMAS* ≥ 2) (HSG *n* = 14) and Low (MMAS < 2) (LSG *n* = 14) spasticity study groups.

	Low spasticity group (*n* = 14)	High spasticity group (*n* = 14)	*p*-value
Timed Up and Go test (seconds)	17.8 ± 9.9	23.4 ± 8.09	0.11
Balance confidence	1051.4 ± 279.9	996.07 ± 287.5	0.61
Reconstruction error angle (degree)	1.14 ± 0.9	2.03 ± 0.9	0.01*
Ankle passive dorsiflextion range of motion (°)	12.2 ± 1.7	11.6 ± 2.6	0.45

Data are presented as means ± SD; *statistically significant at p ≤ 0.05, *MMAS, Modified Modified Ashworth scale.

The distribution of spasticity severity based on MMAS in low and high spasticity groups is presented in Table 3. In the low spasticity group, spasticity severity of five patients were MMAS = 1 and spasticity severity of nine patients were MMAS = 2. In the high spasticity group, spasticity severity of nine patient were MMAS = 3 and spasticity severity of other five patients were MMAS = 4.

**TABLE 3:** Distribution of spasticity of patients based on MMAS* in two groups (*n* = 28).

Group	Subjects	Affected side	MMAS
**Low spasticity group (MMAS < 2)**	1	Left	1
	2	Left	1
	3	Right	2
	4	Left	2
	5	Right	2
	6	Right	2
	7	Left	2
	8	Right	1
	9	Left	1
	10	Right	2
	11	Left	1
	12	Right	2
	13	Right	2
	14	Left	2
**High spasticity group (MMAS ≥ 2)**	1	Right	3
	2	Left	4
	3	Left	4
	4	Left	4
	5	Right	3
	6	Right	3
	7	Right	4
	8	Left	3
	9	Right	4
	10	Right	3
	11	Left	3
	12	Right	3
	13	Right	3
	14	Left	3

*MMAS, Modified Modified Ashworth scale.

### Posturography

Posturography data for the groups are presented in Table 4. There were no significant differences in the medio-lateral (ML) and anterio-posterior (AP) displacements, velocity, and area between open and close eyes conditions, between the affected and unaffected limbs within groups and between groups. The interactions between the groups and the limbs or eyes conditions were not significant (*p* > 0.05).

**TABLE 4:** Posturography data of the High (MMAS* ≥ 2) (HSG** *n* = 14) and Low (MMAS < 2) (LSG*** *n* = 14) spasticity study groups.

			Affected lower limb			Less affected lower limb		
			Mean ± SD	Max	Min	Mean ± SD	Max	Min
Low spasticity group (*n* = 14)	Closed eyes	Anterio-posterior displacement (mm)	0.55 ± 0.32	0.21	1.13	0.50 ± 0.26	0.19	1.2
		Medio-lateral displacement (mm)	0.90 ± 0.32	0.28	1.62	0.84 ± 0.15	0.64	1.14
		Velocity (m.sec^–1^)	1.36 ± 1.30	0.51	5.66	0.88 ± 0.23	0.48	1.41
		Area (m^2^)	2.90 ± 0.42	2.32	3.75	2.83 ± 0.34	3.42	2.33
	Open eyes	Anterio-posterior displacement (mm)	0.69 ± 0.63	0.15	2.54	0.57 ± 0.33	0.22	1.26
		Medio-lateral displacement (mm)	1.10 ± 0.66	0.55	3.25	0.84 ± 0.16	0.63	1.19
		Velocity (m.sec^–1^)	1.26 ± 0.96	0.50	4.37	0.9 ± 0.23	0.69	1.51
		Area (m^2^)	2.90 ± 0.42	2.30	3.83	2.8 ± 0.33	2.34	3.37
High spasticity group (*n* = 14)	Closed eyes	Anterio-posterior displacement (mm)	0.64 ± 0.45	0.21	1.74	0.43 ± 0.26	0.12	0.92
		Medio-lateral displacement (mm)	1.01 ± 0.30	0.41	1.76	0.78 ± 0.27	0.46	1.44
		Velocity (m.sec^–1^)	1.16 ± 0.39	0.69	2.09	0.75 ± 0.29	0.37	1.21
		Area (m^2^)	2.90 ± 0.42	2.30	3.84	2.9 ± 0.33	2.38	3.48
	Open eyes	Anterio-posterior displacement (mm)	0.54 ± 0.42	0.12	1.64	0.52 ± 0.3	0.01	0.97
		Medio-lateral displacement (mm)	1.00 ± 0.32	0.41	1.72	0.73 ± 0.26	0.39	1.27
		Velocity (m.sec^–1^)	1.14 ± 0.37	0.66	2.02	0.76 ± 0.29	0.37	1.22
		Area (m^2^)	2.80 ± 0.45	2.26	3.72	2.9 ± 0.38	2.29	3.53

mm, millimeters; m.sec^–1^, meters per second; m^2^, square meters; *MMAS, Modified Modified Ashworth scale; **HSG, High spasticity group; ***LSG, Low spasticity group.

### Correlations

There was a significant correlation between the ABS scores of balance confidence and the TUG test in the HSG (*r* = −0.55, *p* = 0.04) (Table 5). There were no other significant correlations between the variables.

**TABLE 5:** Correlation between variables in the High (MMAS* ≥ 2) (HSG** *n* = 14) and Low (MMAS* < 2) (LSG*** *n* = 14) spasticity study groups low and high spasticity groups.

		TUG	Balance confidence	Proprioception	Passive dorsi-flexion ROM
Low spasticity group (*n* = 14)	TUG		*r* = −0.39	*r* = -0.2	*r* = −0.11
			*p* = 0.16	*p* = 0.49	*p* = 0.69
	Balance confidence			*r* = −0.03	*r* = 0.09
				*P* = 0.93	*p* = 0.74
	Proprioception				*r* = 0.49
					*p* = 0.07
	Ankle plantarflexor MMAS	*r* = −0.05	*r* = −0.46	*r* = −0.47	*r* = −0.36
		*p* = 0.85	*p* = 0.09	*P* = 0.09	*p* = 0.21
High spasticity group (*n* = 14)	TUG test		*r* = −0.55*	*r* = −0.31	*r* = −0.27
			*p* = 0.04	*p* = 0.27	*p* = 0.37
	Balance confidence			*r* = 0.8	*r* = 0.26
				*p* = 0.76	*p* = 0.36
	Proprioception				*r* = 0.36
					*p* = 0.2
	Ankle plantarflexor MMAS	*r* = 0.16	*r* = 0.04	*r* = 0.09	*r* = 0.32
		*p* = 0.85	*p* = 0.09	*p* = 0.09	*p* = 0.26

TUG, Timed up and go test; *MMAS, Modified Modified Ashworth scale; **HSG, High spasticity group; ***LSG, Low spasticity group; ROM, Range of Motion.

## Discussion

To these authors’ knowledge, this is the first study that evaluated the effects of ankle plantar flexor spasticity severity on balance and determined the relationship between the spasticity severity with ankle proprioception, passive ROM, and balance confidence in post-stroke patients. We found no differences between the LSG and HSG groups in terms of balance confidence, dynamic balance, and ankle dorsiflexion ROM. In addition, postural sway in the open and closed eye conditions was not different in both the LSG and HSG groups for both the less affected and affected limbs. However, ankle joint proprioception in terms of repositioning error angle was better in the LSG compared to the HSG. A relationship was found between TUG scores and balance confidence in the HSG.

In the standing position, the central nervous system keeps an individual’s center of pressure within the base of support (Shumway-Cook and Woollacott, 2007). Therefore, the amount of sway of the pressure center is considered as an indicator of the balance control such that less sways indicate more stability or better balance control (Inness et al., 2015). The findings of the present study showed that there was no difference between ML and AP displacements between the low and high spasticity groups. This finding is similar to that of Rahimzadeh Khiabani et al. (2017) that revealed no differences in ML and AP displacements between the two groups of patients with low and high ankle plantar flexor spasticity. However, Rahimzadeh Khiabani et al. (2017), used only a single force plate and the amount of displacement was the sum of the displacements of both affected and less affected legs. A study used two force plates in patients post-stroke and found no differences in ML and AP displacements of posturography (Singer et al., 2013).

Various investigations have examined the balance control of patients post-stroke in the frontal plane (Geurts et al., 2005; Marigold and Eng, 2006). A review of standing balance in patients with stroke found increased ML sway and impaired balance with asymmetric weight bearing toward the less affected limb (Geurts et al., 2005). In the present study, the absence of a difference in ML displacement between the low and high spasticity groups might have been due to the lack of a difference in the severity of hip and knee spasticity between groups. It may be that ML sway occurs primarily due to the activity of hip adductors and abductors (Winter et al., 1996).

In this study, in line with previous studies (Sosnoff et al., 2010; Rahimzadeh Khiabani et al., 2017), there was no difference in the AP displacement of affected and less affected limbs between the low and high spasticity groups. This may be explained by the fact that the hip and knee movement strategies could be utilized to minimize the ankle movements (Sosnoff et al., 2010). In addition, the compensatory activity of the less affected limb may have played a role in limiting AP displacements of both affected limb and less affected limb observed in this study (Rahimzadeh Khiabani et al., 2017). The differences reported in the AP displacements of patients post-stroke and healthy individuals point to the role of spasticity, regardless of its level, adapted strategies to maintain the balance, and posture stabilization through minimizing the displacements in the frontal and sagittal planes (Marigold et al., 2004; Genthon et al., 2008).

Interestingly, no difference was found in the postural sway between the low and high spasticity groups and between the affected and less affected limbs after the removal of the vision. This was unexpected in that patients post-stroke typically rely on the vision for maintaining their balance (Bonan et al., 2004; Marigold and Eng, 2006). A possible reason for this finding could be that both the low and high spasticity groups used a stiffening strategy by maintaining the knees in extension to increase the stability and balance, regardless of their eyes being open or closed (Mansfield et al., 2013). Further, both groups could also have shifted more weight onto the affected limb in both the eyes open and closed conditions as a further strategy to minimize the AP displacements and thus improving the postural balance (Mansfield et al., 2013).

This study demonstrated that the high spasticity group displayed worse ankle proprioception when compared to the low spasticity group. This finding is in agreement with a previous study that showed proprioception impairment in patients in post-stroke (Carey et al., 1993). This finding could be expected as the high spasticity has been shown to impair the accuracy of deep position sense input (Lee et al., 2010; Gao et al., 2011). While a decreased ankle proprioception in post-stroke patients with spasticity may be postulated that can impair the balance function (Horak et al., 1997; Niam et al., 1999; Tyson et al., 2006), this unexpectedly was not found in the present study. This could be due to the use of an ankle strategy depending on the type of somatosensory input for balance control.

In the present study, in line with a previous study (Rahimzadeh Khiabani et al., 2017), no difference was found in the ABC scores between the low and high spasticity groups. We expected with increasing the severity of muscle spasticity, balance confidence would decrease in post-stroke patients. Nevertheless, a negative relationship between balance confidence and static balance as well as gait in patients post-stroke has been demonstrated (Schinkel-Ivy et al., 2016, 2017). In addition, a higher spasticity level has been associate with recurrent falling in patients post-stroke (Wei et al., 2017).

In this study, dynamic balance, as measured by the TUG, was not different between the low and high spasticity groups. This indicated that regardless of spasticity level, the dynamic balance was impaired in this sample of patients with stroke. However, the scores on the TUG test were worse in the High spasticity group than those in the Low spasticity group (23.4 vs. 17.8). A difference of ∼6 s between the two groups was clinically relevant that indicated that with increasing spasticity level severity the dynamic balance would worsen as demonstrated in previous studies with stroke (Lin et al., 2006; Soyuer and Ozturk, 2007; Sommerfeld et al., 2012). Replicating the study with more patients in the groups will clarify it.

Passive ankle ROM was not different between low and high spasticity groups. A previous study has reported that a higher spasticity was associated with higher limitations in the passive ROM (Li, 2020). In this study, both the low and high spasticity group had significant restricted ankle passive ROM. This suggests that the ankle passive ROM is influenced by ankle muscle spasticity regardless of spasticity intensity. Spasticity, weakness of ankle muscles, and muscle contracture may explain the limited ankle passive ROM in both groups (Mecagni et al., 2000; Li, 2020).

There was a significant negative correlation between the balance confidence and TUG test in the High spasticity group. This finding indicates that the time for the TUG test will be lower with more confidence on balance. However, there was no significant correlation between spasticity and proprioception and passive ROM of the ankle, balance confidence, TUG test, and postural sway in each group. The sample size is very important to examine the correlation in cross-sectional studies. As the number of variables increases, more samples are needed (Schönbrodt and Perugini, 2013). The non-significant correlations obtained for the variables might be due to the small sample size. A study with larger sample of stroke patients of different level of spasticity is required to clarify the size of correlations.

### Limitations

Although the sample size was determined, the number of samples may have been too small to investigate the relationship between clinical data and static and dynamic balance (Schönbrodt and Perugini, 2013). In the present study, both groups of stroke patients had spasticity and there was also no control group. A control group consisting of neurologically healthy people or stroke patients without spasticity will help to more closely examine the effect of spasticity on balance.

## Conclusion

These results show that although stroke patients with spasticity have impaired static and dynamic balance, the severity of spasticity has no effect on the exacerbation of balance control. Therefore, the spasticity post stroke must be considered for management regardless of its severity.

## Data Availability Statement

The raw data supporting the conclusions of this article will be made available by the authors, without undue reservation.

## Ethics Statement

The studies involving human participants were reviewed and approved by the Ethics Committee of Tehran University of Medical Sciences (IR.TUMS.FNM.REC.1397.012). The patients/participants provided their written informed consent to participate in this study.

## Author Contributions

NN, SN, and AM presented the study conception and idea. NN, SN, AM, OM, EG, BS, and IS designed the study protocol. AM drafted the first version of this manuscript that was reviewed and revised critically for intellectual content by NN. AM, EG, and OM collected the data. NN, SN, BS, and IS revised the manuscript. All authors read, commented, and approved the final manuscript for submission.

## Conflict of Interest

The authors declare that the research was conducted in the absence of any commercial or financial relationships that could be construed as a potential conflict of interest.

## Publisher’s Note

All claims expressed in this article are solely those of the authors and do not necessarily represent those of their affiliated organizations, or those of the publisher, the editors and the reviewers. Any product that may be evaluated in this article, or claim that may be made by its manufacturer, is not guaranteed or endorsed by the publisher.
